# Peri- and intra-nodular radiomic features based on ^18^F-FDG PET/CT to distinguish lung adenocarcinomas from pulmonary granulomas

**DOI:** 10.3389/fmed.2024.1453421

**Published:** 2024-08-07

**Authors:** Congna Tian, Yujing Hu, Shuheng Li, Xinchao Zhang, Qiang Wei, Kang Li, Xiaolin Chen, Lu Zheng, Xin Yang, Yanan Qin, Yanzhu Bian

**Affiliations:** ^1^Hebei Medical University, Shijiazhuang, Hebei, China; ^2^Department of Nuclear Medicine, Hebei General Hospital, Shijiazhuang, Hebei, China; ^3^Department of Nuclear Medicine, Affiliated Hospital of Hebei University, Baoding, Hebei, China; ^4^Department of Nuclear Medicine, The Fourth Hospital of Hebei Medical University, Shijiazhuang, Hebei, China

**Keywords:** radiomics, pulmonary granuloma, lung adenocarcinoma, ^18^F-FDG, PET/CT

## Abstract

**Objective:**

To compare the effectiveness of radiomic features based on ^18^F-FDG PET/CT images within (intranodular) and around (perinodular) lung nodules/masses in distinguishing between lung adenocarcinoma and pulmonary granulomas.

**Methods:**

For this retrospective study, ^18^F-FDG PET/CT images were collected for 228 patients. Patients diagnosed with lung adenocarcinoma (*n* = 156) or granulomas (*n* = 72) were randomly assigned to a training (*n* = 159) and validation (*n* = 69) groups. The volume of interest (VOI) of intranodular, perinodular (1–5 voxels, termed Lesion_margin1 to Lesion_margin5) and total area (intra- plus perinodular region, termed Lesion_total1 to Lesion_total5) on PET/CT images were delineated using PETtumor and Marge tool of segmentation editor. A total of 1,037 radiomic features were extracted separately from PET and CT images, and the optimal features were selected to develop radiomic models. Model performance was evaluated using the area under the receiver operating characteristic curve (AUC).

**Results:**

Good and acceptable performance was, respectively, observed in the training (AUC = 0.868, *p* < 0.001) and validation (AUC = 0.715, *p* = 0.004) sets for the intranodular radiomic model. Among the perinodular models, the Lesion_margin2 model demonstrated the highest AUC in both sets (0.883 and 0.616, *p* < 0.001 and *p* = 0.122). Similarly, in terms of total models, Lesion_total2 model was found to outperform others in the training (AUC = 0.879, *p* < 0.001) and validation (AUC = 0.742, *p* = 0.001) sets, slightly surpassing the intranodular model.

**Conclusion:**

When intra- and perinodular radiomic features extracted from the immediate vicinity of the nodule/mass up to 2 voxels distance on ^18^F-FDG PET/CT imaging are combined, improved differential diagnostic performance in distinguishing between lung adenocarcinomas and granulomas is achieved compared to the intra- and perinodular radiomic features alone.

## 1 Introduction

Lung cancer stand as the foremost cause of cancer-related death (1). Non-small cell lung cancer (NSCLC) constituted over 85% of lung cancer cases, and lung adenocarcinomas is the most prevalent subtype (2). The early detection and diagnosis of the localized disease plays a crucial role in the improvement in survival (3). However, pulmonary granulomas often pose a challenge as they can mimic adenocarcinomas radiologically, leading to false-positive diagnoses. Hence, differentiating between malignant and benign lung nodules remains a key challenge for diagnostic radiologists and nuclear medicine physicians.

Radiomics, an emerging field in medical imaging, enables the automatic extraction of high-throughput quantitative features to reveal relationships between image voxels that may elude radiologists’ naked eye. These features can convey biological information such as cell morphology, molecular characteristics, and gene expression. Several studies have shown that radiomics can enhance the diagnostic, prognostic, and predictive accuracy of lung nodules (4–10). However, these studies predominantly focused on intranodular radiomic characteristics like shape, edge, and texture. Conventionally, morphological characteristics such as spiculation and structural distortion of the surrounding parenchyma are used to evaluate malignant lung nodules on CT images. The perinodular parenchyma holds biological significance concerning cell migration, inflammation, and vascularization. Considering that benign and malignant lung nodules interact differently with the surrounding parenchyma, radiomic features reflecting heterogeneity patterns in the immediate vicinity outside the nodule may have potential predictive value for malignancy. Previous studies have demonstrated that incorporating perinodular parenchymal features on CT images can improve lung nodule classification (11–16).

CT images offer high-resolution anatomical details, including speculation, lobulation, vessel convergence, and air bronchogram, while PET images capture glucose metabolism information. ^18^F-FDG PET/CT imaging has become an essential modality for diagnosing, staging, evaluating therapy response, and predicting prognosis for NSCLC (17). Despite this, there have been relatively few studies on PET/CT intranodular radiomic features to differentiate between tuberculosis and lung cancer, Hu et al. (6) and Du et al. (18) found that PET/CT radiomic features achieved an AUC of 0.861 and 0.97, respectively, suggesting potential for lung nodule classification. Du et al. (18) observed that the PET/CT-derived features improved diagnostic performance compared to CT-only signatures. However, to date, no studies have been conducted on ^18^F-FDG PET/CT-derived perinodular radiomic features to differentiate between lung adenocarcinomas and pulmonary granulomas. Our study aims to evaluate whether ^18^F-FDG PET/CT radiomic features associated with perinodular parenchyma can predict lung adenocarcinomas, and whether combining peri- and intranodular radiomic patterns enhances predictive accuracy compared to intranodular determination alone.

## 2 Materials and methods

### 2.1 Patients

In this retrospective diagnostic study, patients with a solitary pulmonary nodule/mass were recruited from Hebei General Hospital between April 2014 and June 2023. The institutional ethics committee approved the retrospective analysis of all data and waived the requirement for informed consent. Patients with the following criteria were included: (1) confirmed lung adenocarcinoma or granulomas (e.g., inflammatory pseudotumor, tuberculosis, or organizing pneumonia) via surgery, biopsy, or follow-up; (2) ^18^F-FDG PET/CT scan conducted prior to surgery or biopsy; (3) absence of other malignant tumors history; (4) lesion with a maximum diameter of ≥ 8 mm. According to Chinese expert consensus on diagnosis of early lung cancer (2023 Edition), PET/CT can be used to differentiate between benign and malignant pulmonary nodules with a diameter of ≥ 8 mm (19). The criterion for exclusion were: (1) lesions lacking ^18^F-FDG metabolism; (2) non-solitary pulmonary nodules/masses; and (3) PET images displaying respiratory motion artifact.

**FIGURE 1:**
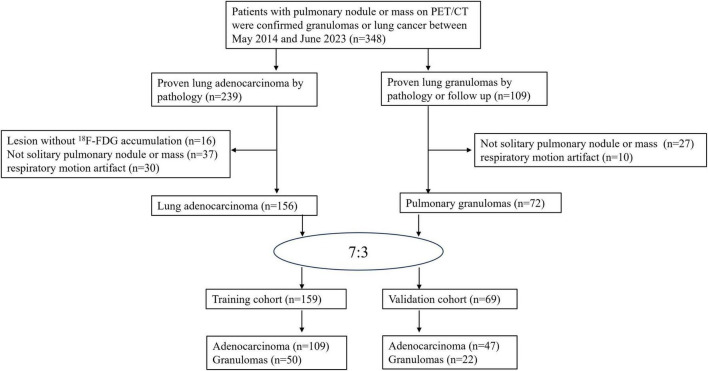
Flowchart for the patient selection process.

Based on the aforementioned criteria, the final cohort comprised 228 patients (133 males and 95 females) with an average age of 64.12 ± 11.10 years (range 21–90). Patients were randomly allocated to a training set comprising 159 patients (109 adenocarcinomas, 50 granulomas), and a validation set comprising 47 adenocarcinomas and 22 granulomas. The patient selection process is depicted in Figure 1.

### 2.2 PET/CT examination

Patients underwent a minimum 6-h fast before intravenous injection of 3.7–5.55 MBq/kg ^18^F-FDG, followed by a period of rest. Subsequently, a PET/CT scan (Discovery Elite PET/CT; GE Healthcare) was conducted 60 ± 5 min later in accordance with European Association of Nuclear Medicine guidelines (20). CT images were acquired with a tube voltage of 120 kV, 130–280 mA, and slice thickness of 3.75 mm. PET data were collected for 2 min per bed with a matrix size of 192 × 192 in 3D acquisition mode and underwent attenuation correction based on CT images. The attenuation-corrected PET images were reconstructed using iterative reconstruction algorithm (2 iterations and 24 subsets).

### 2.3 Nodule/mass segmentation

**FIGURE 2:**
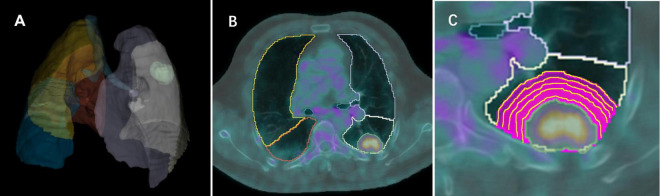
Images show intra- and perinodular segmentation. **(A)** Lung tissue segmentation: a deep learning method (The TotalSegmentator AI model) was used to automatically segment the anatomical structure of the chest in CT images and remove the extrapulmonary structure, **(B)** PETtumor was used to semi-automatically delineate the original 3D volume of interest (VOI) of lung nodule based on PET images, **(C)** Marge tool of segmentation editor was used to grow from the VOI of the nodular edge by 1, 2, 3, 4, 5 voxels, and the nodular segmentation was subtracted to achieve the perinodular segmentation. Intersect the expanded portion with the anatomical structure segmented by CT images to obtain the expanded portion in lung tissue.

PET and CT images in DICOM format were imported into open-source software (3D Slicer, version 5.0)^1^ and registered using General Elastix (with fixed CT) (21). The voxel size of PET and CT images was 0.9766 × 0.9766 × 3.27 mm. A deep learning method (The TotalSegmentator AI model) was used to automatically segment the anatomical structure of the chest in CT images and remove the extrapulmonary structure (22). The nuclear medicine physician with 9 years of experience and a senior with 11 years of experience blinded to the pathologic diagnosis, utilized PETtumor to semi-automatically delineate the volume of interest (VOI) of lung nodules/masses (termed Lesion) based on PET images (23). Marge tool of segmentation editor was then employed to expand from the nodular edge on PET images by 1–5 voxels (marked as Lesion_total1 to Lesion_total5), and the nodular segmentation was subtracted to obtain the perinodular segmentation (named as Lesion_margin1 to Lesion_margin5). The expanded portion within lung tissue was obtained by intersecting the expanded portion with the anatomical structure segmented by CT images. Due to the high correspondence between PET and CT images, features were extracted from both types of images within the same VOI of the intra- and perinodular regions. In cases where there was poor registration between PET and CT images, manual adjustment was performed to ensure excellent alignment between the two image types. Nodule/mass segmentation process is shown in Figure 2.

### 2.4 Radiomic feature extraction and selection

Radiomics features were extracted using Pyradiomics Version 3.1.0, an open-source Python package designed for extracting radiomic features from 2-dimensional and 3-dimensional images and binary masks. All image processing and radiomic feature extraction procedures adhered to the IBSI reporting guidelines (24). PET/CT images underwent processing using the Laplacian of Gaussian (LoG) algorithm (parameters: σ = 2.0, 3.0) and wavelet transform algorithm, then discretized to fixed bin widths of 0.5 for PET and 25 HU for CT, respectively. A total of 1,037 radiomic features were automatically extracted based on the aforementioned volume of interest (VOI), covering eight categories: first-order statistics, shape-based (2D and 3D), gray level co-occurrence matrix (GLCM), gray level dependence matrix (GLDM), gray level run length matrix (GLRLM), gray level size zone matrix (GLSZM), and neighboring gray tone difference matrix (NGTDM). Z-score normalization was applied to standardize radiomic parameters for all patients. Informative features for lung nodule prediction were selected using correlation analysis and general-univariate analysis with or without Gradient Boosting Decision Tree (GBDT) in both training and validation cohorts (25).

### 2.5 Model establishment and testing

In the training cohort, three machine learning models were established using the logistic regression algorithm: the intranodular model, perinodular model and total model. The parameters of these models included optimal radiomic features with significant differences extracted from the intranodular, perinodular, and intra-plus peri-nodular VOIs, respectively. Subsequently, data from the testing set were utilized to test the performance of these models. The predictive performance was evaluated using receiver operating characteristic (ROC) curves and the area under the ROC curve (AUC) in both the training and validation sets.

### 2.6 Statistical analysis

A Student’s *t*-test was used for continuous variables with a normal distribution, while Mann-Whitney test was used for continuous variables with abnormal distribution. Nominal variables were analyzed using either a chi-square test or Fisher’s exact test. ROC curves were used to evaluate the models, and pairwise comparisons of the ROC curves were conducted using DeLong tests. Data handling, model establishment, statistical analysis, and model evaluation were carried out using Python (version 3.5.6), SPSS version 25.0, R (version 3.5.1), and MedCalc software 20.217. A two-tailed *p*-value < 0.05 indicated statistical significance.

## 3 Results

### 3.1 Clinical characteristics

The characteristics of the 228 patients in the training and validation sets are summarized in Table 1. There were no significant differences observed in age (*p* = 0.671), sex (*p* = 0.270), smoking history (*p* = 0.254), carcinoembryonic antigen (CEA) level (*p* = 0.100), cytokeratin 19 fragment (Cyfra 21-1) (*p* = 0.090), the distribution of lobar locations (*p* = 0.173), the type of nodules (*p* = 0.142), or nodular size (*p* = 0.188) between the training and validation cohorts. However, sex exhibited a statistically significant difference between the adenocarcinoma and granuloma groups in both the training (*p* = 0.016) and validation cohorts (*p* = 0.030). CEA levels in the adenocarcinoma subset were significantly higher than that in the granuloma subset in both the training (*p* < 0.001) and validation cohorts (*p* < 0.001). Similarly, Cyfra 21-1 levels were notably elevated in the adenocarcinoma subset compared to the granuloma subset in the training cohort (*p* < 0.001), though no statistical difference was found in the validation cohort (*p* = 0.071). Regarding age (*p* = 0.145 and 0.844) and smoking history (*p* = 0.230 and 0.345), there were no significant differences observed between the adenocarcinoma and granuloma groups in either cohort. The distribution of lobar locations, predominately in the upper lobes, the type of nodules, with more solid nodules, and nodular size were similar between the malignant and benign nodules in both the training and validation sets (all *p* > 0.05). Univariate analyses revealed that CEA level (OR = 2.105, 95% CI [1.310–3.381], *p* = 0.002) and Cyfra 21-1 level (OR = 1.737, 95% CI [1.001–3.014], *p* = 0.049) were significant predictors of lung adenocarcinoma.

**TABLE 1:** Characteristics of patients in the training and validation cohorts.

Characteristic	Training cohort (*n* = 159)			Validation cohort (*n* = 69)		
	Adenocarcinomas (*n* = 109)	Granulomas (*n* = 50)	*P*	Adenocarcinomas (*n* = 47)	Granulomas (*n* = 22)	*P*
Age (mean ± SD, years)	64.93 ± 9.72	61.68 ± 14.48	0.145	64.77 ± 9.42	64.23 ± 12.77	0.844
Sex, *n* (%)			0.016			0.030
Male	54 (49.54)	35 (70.00)		34 (72.34)	10 (45.45)	
Female	55 (50.46)	15 (30.00)		13 (27.66)	12 (54.55)	
Smoking status, *n* (%)			0.230			0.345
Yes	20 (18.35)	11 (22.00)		16 (34.04)	4 (18.18)	
Mean pack-years	14.25 ± 29.49	16.54 ± 30.55	0.730	17.97 ± 22.32	8.33 ± 15.08	0.083
No	54 (49.54)	17 (34.00)		19 (40.43)	11 (50.00)	
Unavailable	35 (32.11)	22 (44.00)		12 (25.53)	7 (31.82)	
CEA, M (IQR)	5.72 (2.63, 11.80)	2.37 (1.54, 2.97)	< 0.001	4.54 (2.85, 30.32)	2.31 (1.43, 2.90)	< 0.001
Cyfra 21-1 (mean ± SD)	3.92 ± 2.92	2.28 ± 0.89	< 0.001	5.11 ± 4.25	2.37 ± 0.74	0.071
Location, *n* (%)			0.581			0.536
Upper and middle	76 (69.72)	37 (74.00)		36 (76.60)	19 (86.36)	
Lower	33 (30.28)	13 (26.00)		11 (23.40)	3 (13.64)	
Type of nodule, *n* (%)			0.142			0.801
Solid nodule	88 (80.73)	45 (90.00)		35 (74.47)	17 (77.27)	
Subsolid nodule	21 (19.27)	5 (10.00)		12 (25.53)	5 (22.73)	
Lesion size (mm)	25.47 ± 7.54	24.06 ± 9.73	0.318	28.79 ± 10.68	25.32 ± 15.23	0.278

SD, standard deviation; M, median; IQR, Interquartile range; CEA, carcinoembryonic antigen; Cyfra 21-1, cytokeratin 19 fragment.

### 3.2 Calculation of the RAD-score

Inter-observer reproducibility of segmentation had been assessed, indicating a good agreement with the correlation coefficients (ICCs) > 0.75. A total of 19 features were screened from the intranodular region to build the radiomics signature in the training cohort. The radiomics signature score (RAD-score) for each enrolled patient was computed using the following logistic regression formula:

RAD-score = 1.178984–0.211528 × original_shape_Elongation + 0.347640 × original_shape_ SphericityCT + 0.113382 × original_firstorder_90Percentile–0.230377 × original_ NGTDM_ Complexity + 0.477789 × log-sigma-2-0-mm-3D_ firstorder_Minimum + 0.167416 × log-sigma-3-0-mm-3D_ GLDM_LargeDependenceLowGrayLevelEmphasis–0.291020 × log-sigma-3-0-mm-3D_ GLSZM_GrayLevelVariance + 0.011914 × log-sigma-3-0-mm-3D_GLSZM_SmallAreaEmphasis + 0.549300 × wavelet-HLL_GLDM_LargeDependenceLowGrayLevelEmphasis–0.082842 × wavelet-HHL_GLCM_Correlation + 0.089530 × wavelet-HHH_GLSZM_GrayLevelNonUniformityNormalized + 0.347640 × original_shape_Sphericity + 0.418771 × original_ firstorder_Minimum–0.452291 × wavelet-LLH_firstorder_ Skewness + 0.095162 × wavelet-LLH_GLCM_InverseVariance–0.171994 × wavelet-LLH_GLDM_DependenceVariance + 0.500768 × wavelet-LHL_GLCM_InverseVariance −0.316888 × wavelet-LHL_GLCM_MaximumProbability–0.794517 × wavelet-LHL_NGTDM_ Busyness.

Among these features, the first 11 are derived from CT, while the last 8 are PET features. The calculation formulas for RAD-score of perinodular models and total models are provided in electronic Supplementary material.

The median and interquartile range of the calculated RAD-score from all regions are presented in Table 2. The RAD-score from intranodular, perinodular or total regions in the lung adenocarcinoma group was significantly higher than that in the granuloma group in the training cohort, and there was a significant difference between the two subsets (all *p* < 0.001). In the validation cohort, lung adenocarcinomas exhibited higher RAD-score than the granuloma group from intranodular or total regions (intranodular plus perinodular distance of 2–4 voxels outside the nodule) (all *p* < 0.05). However, there was no statistical difference between the two groups from perinodular or total regions (intranodular plus perinodular distance of 1/5 voxels outside the nodule) (all *p* > 0.05).

**TABLE 2:** Comparison of radiomic signature score (RAD-score) between lung adenocarcinoma subset and pulmonary granuloma subset in training cohort and validation cohort.

Characteristic	Training cohort		*P*	Validation cohort		*P*
	Adenocarcinoma	Granulomas		Adenocarcinoma	Granulomas	
Lesion	1.897 (1.054, 3.182)	−0.313 (−1.840, 0.748)	< 0.001	1.558 (0.480, 3.086)	0.166 (−1.718, 2.111)	0.004
Lesion_margin1	2.188 (0.889, 3.393)	−0.429 (−1.421, 0.564)	< 0.001	1.667 (1.093, 2.364)	1.668 (0.298, 2.171)	0.709
Lesion_margin2	2.329 (0.874, 3.856)	−0.852 (−1.645, 0.706)	< 0.001	1.161 (0.334, 2.462)	0.672 (−0.336, 1.618)	0.122
Lesion_margin3	2.388 (0.823, 3.628)	−0.558 (−1.627, 0.521)	< 0.001	1.224 (0.218, 2.159)	1.154 (−0.131, 1.720)	0.520
Lesion_margin4	1.685 (0.759, 3.020)	−0.416 (−1.081, 0.739)	< 0.001	0.884 (0.609, 2.118)	1.307 (0.331, 1.940)	0.898
Lesion_margin5	1.345 (0.541, 2.104)	0.389 (−0.334, 0.864)	< 0.001	1.064 (0.400, 1.514)	0.801 (0.320, 1.582)	0.787
Lesion_total1	2.295 (1.232, 3.009)	−0.763 (−2.156, 0.756)	< 0.001	0.665 (−0.475, 2.437)	1.831 (−0.821, 4.062)	0.285
Lesion_total2	2.077 (0.917, 3.181)	−0.559 (−1.788, 0.288)	< 0.001	2.020 (0.651, 2.896)	−0.228 (−1.234, 1.148)	0.001
Lesion_total3	2.209 (0.994, 3.902)	−0.809 (−1.874, 0.326)	< 0.001	1.511 (0.429, 4.222)	−0.623 (−2.185, 2.735)	0.015
Lesion_total4	1.613 (0.868, 2.548)	−0.280 (−1.606, 0.844)	< 0.001	1.873 (0.729, 2.518)	0.743 (−0.802, 1.442)	0.004
Lesion_total5	1.667 (0.777, 2.638)	−0.944 (−1.679, 1.244)	< 0.001	1.640 (−0.016, 2.596)	−0.140 (−0.877, 2.334)	0.160

The value of RAD-score was expressed as median (interquartile range).

### 3.3 Intra- and perinodular features to distinguish adenocarcinomas from granulomas

**FIGURE 3:**
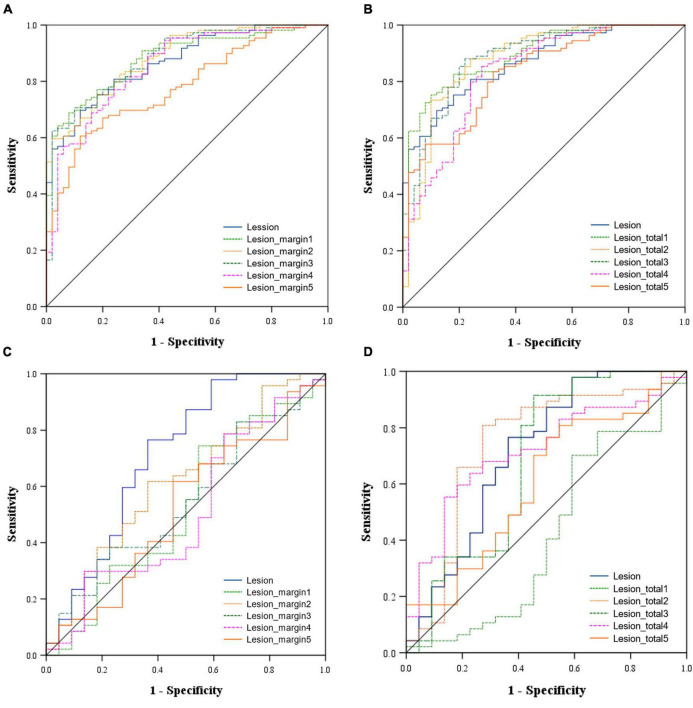
Area under the receiver operating characteristic (ROC) curve of the models in the training cohort [**(A)** Intranodular and perinodular models, **(B)** Intranodular and total models and in the validation cohort **(C)** Intranodular and perinodular models, **(D)** Intranodular and total models].

The predictive performance of the 11 models in both the training and validation cohorts is summarized in Table 3, with corresponding ROC curves illustrated in Figures 3A–D. The intranodular radiomic model demonstrated strong performance in both the training (AUC: 0.868, *p* < 0.001) and validation (AUC: 0.715, *p* = 0.004) cohorts. Among the perinodular models, the Lesion_margin2 model exhibited the highest AUC values (AUC: 0.883 and 0.616, *p* < 0.001 and *p* = 0.122) in both cohorts, although the AUC of the validation set is not statistically significant. Similarly, in terms of total models, the Lesion_total2 model showed the highest AUC in both the training (0.879, *p* < 0.001) and validation (0.742, *p* = 0.001) sets, outperforming the intranodular model.

**TABLE 3:** AUC values obtained in the training and validation cohorts by using different feature extraction from intra- and perinodular region to distinguish adenocarcinomas from granulomas on PET/CT images.

Intranodular model			Perinodular model		Total model	
Training AUC	Validation AUC		Training AUC	Validation AUC	Training AUC	Validation AUC
0.868 (0.823, 0.911)	0.715 (0.585, 0.833)	1 voxel	0.881 (0.835, 0.921)	0.528 (0.391, 0.659)	0.895 (0.850, 0.932)	0.420 (0.290, 0.558)
		2 voxels	0.883 (0.838, 0.924)	0.616 (0.487, 0.734)	0.879 (0.825, 0.927)	0.742 (0.620, 0.854)
		3 voxels	0.880 (0.829, 0.924)	0.548 (0.428, 0.669)	0.885 (0.833, 0.932)	0.682 (0.545, 0.815)
		4 voxels	0.852 (0.798, 0.904)	0.510 (0.381, 0.639)	0.833 (0.773, 0.890)	0.718 (0.606, 0.817)
		5 voxels	0.780 (0.719, 0.838)	0.520 (0.394, 0.647)	0.830 (0.774, 0.883)	0.605 (0.48, 0.727)

Total model includes intra- and perinodular features. Data in parentheses are 95% confidence intervals.

The DeLong test revealed that in the training set, the AUC of the Lesion_margin5 model was significantly lower than those of other perinodular models and the intranodular model (all *p* < 0.05). However, no significant difference was found between other perinodular models and the intranodular model (all *p* > 0.05). In the validation set, the AUC of the intranodular model was statistically higher than those of perinodular models (all *p* < 0.05), except for the Lesion_margin2 model (*p* > 0.05). Among the total models and intranodular model, a significant difference between the AUC of the Lesion_total1 model and that of the Lesion_total5 model (*p* = 0.0289) was detected, but this difference was not observed in other models (all *p* > 0.05) in the training set. In the validation set, the AUC of the Lesion_total5 model was significantly lower than that of the Lesion_total2 model (*p* = 0.0293) and Lesion_total4 model (*p* = 0.0453), while no significant difference was found between other models (all *p* > 0.05).

## 4 Discussion

This study initially assessed the ability of radiomic features extracted from both the segmented nodule/mass alone (intranodular region) and perinodular region on ^18^F-FDG PET/CT images, defined as the immediate lung parenchyma outside the nodule up to the distance of 1 to 5 voxels, in distinguishing between lung adenocarcinomas and granulomas. We found that the gross radiomics signature from the intranodular region and the immediate vicinity extending to 2 voxels outside the nodule yielded the most favorable performance (AUC = 0.879). Our findings indicated that incorporating perinodular radiomic features alongside the intranodular features enhanced the predictive capability of the approach for lung cancer in both the training and validation sets, although the observed differences were not highly significant. The proposed model serves as a non-invasive diagnostic tool for discriminating between granuloma nodules and tumor nodules, potentially reducing the necessity for invasive diagnostic procedures.

The majority of radiomic studies focusing on distinguishing between malignant and benign lung nodules have primarily utilized features derived solely from the nodule itself. However, in recent years, there has been increasing interest in exploring the integration of CT perinodular features from the surrounding parenchyma for lung nodule classification. Dilger et al. (15) showcased the potential of perinodular parenchymal signals manually segmented from CT data to enhance lung nodular classification in a cohort of 50 subjects. They found that nodule-only features achieved an AUC of 0.918 (including nodule size) and 0.872 (excluding nodule size), the inclusion of parenchymal features led to improved performance (AUC of 0.938). Although these results supported the hypothesis of differential influence of malignant versus benign nodules on perinodular pulmonary parenchyma, they lacked an independent validation set. Similarly, Beig et al. (11) demonstrated that incorporating perinodular radiomic features from the immediate vicinity of 5 mm outside the lung nodule to intranodular features in CT imaging enhanced the predictive ability of distinguishing adenocarcinomas from granulomas, yielding an AUC of 0.80. They further correlated histologic features of adenocarcinoma and granuloma with radiomic features to gain insight into the biological underpinnings of their findings. Perinodular regions may serve as the tumor microenvironment, which plays a critical role in defining biological behavior such as aggressiveness, metastatic potential, and therapy response in oncology. Building upon the tumor microenvironment concept, Beig et al. proposed that the perinodular zone or habitat of a malignant lesion may exhibit distinct molecular, cellular, or radiological alterations compared to a benign lesion. Histologically, densely packed tumor-infiltrating lymphocytes and tumor-associated macrophages were observed around adenocarcinomas, whereas giant cells comprising histiocytes and macrophages were noted at the interface of granuloma and normal lung tissue, potentially explaining the diverse machine-extracted radiomic features between the two diseases. However, it is noteworthy that their study solely extracted two-dimensional features from a single representative slice, and the utilization of different types of CT scanners with varying section thicknesses and reconstruction methods may have limited the robustness of the results.

Our study extracts three-dimensional radiomic features of nodules/masses and perinodular region from volumetric segmentation, also takes PET-based radiomic features into account, resulting in improved diagnostic performance with higher AUC values. Previous investigations (26) have established that the heterogeneity of FDG uptake on PET images reflects the distribution of various tissue components within the primary tumor, such as cell infiltration, abnormal angiogenesis, myxoid changes, and necrosis. Peritumoral tissues may indeed harbor infiltrative tumor cells or lymphocytes, which manifest as FDG uptake on PET images. Additionally, slight FDG uptake in the perinodular region might arise from the partial volume effect (27). The partial volume effect, particularly concerning small nodules, can significantly impact the accuracy of PET measurements by causing underestimation of radiotracer uptake in small lesions due to limited spatial resolution. In this study, except for one patient with a pulmonary nodule diameter of 8 mm, all other lesions were over 1 cm in size. The partial volume effect had a relatively small impact on the measurement of glucose uptake in the lesions and the establishment of radiomics models.

In most previous studies, the region of interest used in the perinodular zone segmentation algorithms was defined with a fixed size. Uthoff et al. (14) investigated the utility of machine learning tools incorporating perinodular parenchymal features (parenchyma quartile bands: 25, 50, 75, and 100% of the maximum in-plane diameter of the nodule) for distinguishing between malignant and benign lung nodules. Their results demonstrated that the four machine learning tools incorporating parenchymal signals outperformed exclusively nodular features, with no statistical difference observed between the tools including parenchymal features. Similarly, our study evaluated the significance of radiomic features from different areas surrounding pulmonary nodules. We found that the inclusion of perinodular features from the region immediately adjacent to the nodule up to 2 voxels (approximately 6.5 mm) distance exhibited superior performance compared to nodular-only features. The discrepancies in results may be attributed to differences in segmentation methods for the surrounding area of the nodule and the integration of PET image information. The areas yielding the most meaningful perinodular features extracted in our study align with findings reported by Beig et al. (11) and Lin et al. (16) (morphological expansion of 5 mm).

In our study, the semi-automatic delineation of VOI for intranodular and perinodular regions serves to reduce the workload of radiologists, enhancing reproducibility and stability compared to manual delineation methods.

However, it is important to acknowledge several limitations in our study. Firstly, the cohort comprised 228 patients with an imbalanced ratio of adenocarcinomas to granulomas, nearly 2:1. It is plausible that with a more balanced dataset including a greater number of benign nodules, the performance could be improved. Secondly, our study may have been underpowered to demonstrate statistically significant improvements in the resulting AUC values of the total model, due to the limited number of representative examples, and its single-center retrospective design. A prospective study involving larger multi-center patient cohorts is warranted to further validate the reproducibility and reliability of machine learning models, thereby facilitating the integration of this noninvasive and convenient method into clinical practice. Furthermore, it is imperative to investigate intra- and inter-observer variabilities of the features in future studies to ensure the generalizability of the model. Finally, we intend to augment the machine learning model by incorporating clinical features and subjective radiological features to enhance the accuracy of the model.

## 5 Conclusion

Our study demonstrates that combining intranodular and perinodular radiomic features extracted from the immediate vicinity of the nodule/mass up to 2 voxels distance on ^18^F-FDG PET/CT imaging leads to improve the performance in distinguishing between lung adenocarcinomas and granulomas. These findings underscore the significance of incorporating perinodular features in the classification of solid pulmonary nodules/masses. The proposed model offers a noninvasive and promising auxiliary diagnostic tool that holds potential for integration into routine clinical practice in the future, so that minimizing the need for repeated CT or PET/CT imaging and reducing radiation exposure for benign nodules, and accelerating the treatment process for malignant nodules.

## Data availability statement

The raw data supporting the conclusions of this article will be made available by the authors, without undue reservation.

## Ethics statement

The studies involving humans were approved by the Ethics Committee of Hebei General Hospital. The studies were conducted in accordance with the local legislation and institutional requirements. The ethics committee/institutional review board waived the requirement of written informed consent for participation from the participants or the participants’ legal guardians/next of kin because the cases used in this study were obtained from previous clinical practices.

## Author contributions

CT: Conceptualization, Data curation, Formal analysis, Funding acquisition, Investigation, Methodology, Software, Writing – original draft. YH: Conceptualization, Funding acquisition, Project administration, Supervision, Validation, Writing – review & editing. SL: Data curation, Software, Validation, Writing – review & editing. XZ: Data curation, Software, Validation, Writing – review & editing. QW: Data curation, Validation, Writing – review & editing. KL: Data curation, Validation, Writing – review & editing. XC: Data curation, Validation, Writing – review & editing. LZ: Data curation, Validation, Writing – review & editing. XY: Data curation, Validation, Writing – review & editing. YQ: Data curation, Validation, Writing – review & editing. YB: Conceptualization, Project administration, Supervision, Validation, Writing – review & editing.

## Footnotes

1
https://www.slicer.com
