# Supplementary material for: Peri- and intra-nodular radiomic features based on 18F-FDG PET/CT to distinguish lung adenocarcinomas from pulmonary granulomas
Source: Front Med (Lausanne). 2024 Aug 7;11:1453421. doi: 10.3389/fmed.2024.1453421 (PMC11339787; doi:10.3389/fmed.2024.1453421)
Supplement: Supplementary file 1 [file Data_Sheet_1.PDF]

Journal: Frontiers in Medicine

Supplementary material caption: The calculation formula for Rad-score of perinodular models and total models

Characteristic	Formula for Rad-score	Source of features
Lesion_margin1	1.40097+0.299946*log-sigma-3-0-mm-3D_GLCM_ClusterShade+0.028657*wavelet-LHL_firstorder_Maximum-0.841093*wavelet-HLL_firstorder_Kurtosis-0.263937*wavelet-HLL_GLCM_Correlation-0.286386*wavelet-HLL_GLCM_MCC+0.14492*wavelet-HLH_GLCM_Correlation-0.000965*wavelet-LLL_GLDM_SmallDependenceLowGrayLevelEmphasis+0.679347*original_firstorder_Minimum-0.88703*log-sigma-2-0-mm-3D_GLCM_ClusterTendency+0.972737*wavelet-LLH_GLCM_ClusterShade+0.527676*wavelet-HLL_GLCM_ClusterShade+0.225445*wavelet-HLH_NGTDM_Contrast+0.711792*wavelet-HHH_firstorder_Skewness-0.07063*wavelet-HHH_GLCM_Imc2	the first 7derived from CT, and the last 7 are PET features
Lesion_margin2	1.541233-0.445265*original_GLDM_SmallDependenceLowGrayLevelEmphasis-0.682673*original_GLSZM_LargeAreaLowGrayLevelEmphasis+0.265311* wavelet-LHL_firstorder_Mean-0.106765*wavelet-LHH_firstorder_Maximum-0.913531*wavelet-HLL_GLCM_MCC+0.216508*wavelet-HLH_firstorder_Kurtosis+0.683816*original_firstorder_Minimum-1.028324*log-sigma-2-0-mm-3D_GLCM_Imc2+0.237038*log-sigma-2-0-mm-3D_GLSZM_ZonePercentage+1.091281*log-sigma-3-0-mm-3D_NGTDM_Coarseness+1.441872*wavelet-LLH_GLCM_ClusterShade-0.336345*wavelet-LHL_firstorder_InterquartileRange+0.273927*wavelet-HLL_firstorder_InterquartileRange-0.624926*wavelet-HLL_GLCM_MaximumProbability+0.412226*wavelet-LLL_GLSZM_SizeZoneNonUniformityNormalized	the first 6 derived from CT, and the last 9 are PET features
Lesion_margin3	1.635368-0.355808*original_GLSZM_LargeAreaHighGrayLevelEmphasis-0.34288*wavelet-LLH_GLCM_Idmn+0.06826*wavelet-LHL_firstorder_Mean+0.078274*wavelet-HHH_GLCM_InverseVariance-0.487912*wavelet-LLL_GLSZM_LargeAreaLowGrayLevelEmphasis+0.875051*original_firstorder_Minimum-0.801809*log-sigma-2-0-mm-3D_GLCM_Imc2+1.46225*wavelet-LLH_GLCM_ClusterShade+0.487542*wavelet-LHH_firstorder_Median+0.799237*wavelet-HLL_firstorder_Mean+1.149579*wavelet-HHL_GLSZM_SizeZoneNonUniformityNormalized+0.243421*wavelet-HHH_firstorder_Variance-0.387492*wavelet-HHH_GLCM_ClusterShade	the first 5 derived from CT, and the last 8 are PET features
Lesion_margin4	1.46834-0.064297*log-sigma-3-0-mm-3D_GLCM_Idmn-0.134275*wavelet-LLH_GLCM_Idmn+0.108295*wavelet-LHL_firstorder_Mean-0.301033*wavelet-HHH_firstorder_Mean+0.058637*wavelet-HHH_GLCM_InverseVariance-0.609418*wavelet-LLL_GLSZM_LargeAreaHighGrayLevelEmphasis-0.529226*wavelet-LLL_GLSZM_LargeAreaLowGrayLevelEmphasis+0.612902*original_firstorder_Minimum-0.065517*original_GLSZM_LargeAreaLowGrayLevelEmphasis+0.007889*log-sigma-3-0-mm-3D_GLCM_ClusterShade+0.043693*log-sigma-3-0-mm-3D_GLSZM_SizeZoneNonUniformityNormalized+0.05833*log-sigma-3-0-mm-3D_GLSZM_ZonePercentage+2.472267*wavelet-LLH_GLCM_ClusterShade-0.625832*wavelet-HLH_GLCM_MaximumProbability+0.516719*wavelet-HHH_firstorder_Skewness-0.483996*wavelet-HHH_GLCM_ClusterShade-0.460841*wavelet-HHH_GLCM_SumSquares	the first 7 derived from CT, and the last 10 are PET features
Lesion_margin5	1.30667+0.196758*wavelet-HHH_GLCM_InverseVariance-0.189566*wavelet-HHH_GLSZM_LargeAreaLowGrayLevelEmphasis-0.309947*wavelet-LLL_GLSZM_LargeAreaHighGrayLevelEmphasis-0.410941*wavelet-LLL_GLSZM_LargeAreaLowGrayLevelEmphasis+0.576372*original_firstorder_Minimum+2.289769*wavelet-LLH_GLCM_ClusterShade-0.05197*wavelet-LLH_NGTDM_Busyness-0.140724*wavelet-HLL_firstorder_Uniformity+0.444342*wavelet-HHH_firstorder_Median	the first 4 derived from CT, and the last 5 are PET features

Characteristic	Formula for Rad-score	Source of features
Lesion_total1	1.249162+0.395916*original_shape_Sphericity-0.389972*log-sigma-2-0-mm-3D_firstorder_Minimum+0.118035*log-sigma-3-0-mm-3D_GLRLM_LongRunHighGrayLevelEmphasis-0.549838*wavelet-LLH_GLCM_ClusterShade+0.272785*wavelet-HLL_GLCM_MCC-0.943171*wavelet-HHH_GLCM_Correlation-0.255505*wavelet-HHH_GLCM_InverseVariance-1.027367*wavelet-LLL_firstorder_10Percentile+0.060078*original_firstorder_Minimum+0.769547*original_GLSZM_SizeZoneNonUniformityNormalized+0.015446*log-sigma-3-0-mm-3D_NGTDM_Busyness-0.732488*wavelet-LLH_GLDM_DependenceVariance+0.391845*wavelet-LHL_GLCM_InverseVariance-0.496341*wavelet-LHL_NGTDM_Busyness-0.465324* wavelet-HLL_GLCM_MaximumProbability-0.024288*wavelet-HLH_firstorder_Mean+0.083612*wavelet-HHL_GLDM_DependenceEntropy	the first 8 derived from CT, and the last 9 are PET features
Lesion_total2	1.384462+0.142356*log-sigma-3-0-mm-3D_GLDM_LargeDependenceLowGrayLevelEmphasis-0.652277*wavelet-LLH_firstorder_Median+0.209779*wavelet-LLH_GLCM_ClusterShade+0.215397*wavelet-LHH_GLSZM_SizeZoneNonUniformityNormalized-0.542526*wavelet-HLL_firstorder_Kurtosis-0.476824*wavelet-HHH_GLCM_Correlation+0.781289*original_firstorder_Minimum+0.835516*log-sigma-2-0-mm-3D_GLSZM_GrayLevelNonUniformity+0.048287*wavelet-LHL_GLRLM_ RunLengthNonUniformityNormalized+0.175046*wavelet-LHL_NGTDM_Strength-1.165557*wavelet-LHH_firstorder_Mean-0.266764*wavelet-HLL_GLCM_MaximumProbability-1.017663*wavelet-HLL_GLSZM_LargeAreaLowGrayLevelEmphasis	the first 6 derived from CT, and the last 7 are PET features
Lesion_total3	1.475445+0.971756*log-sigma-3-0-mm-3D_GLRLM_LowGrayLevelRunEmphasis+0.45228*wavelet-LLH_GLCM_ClusterShade+0.379753*wavelet-LHH_GLSZM_SizeZoneNonUniformityNormalized-0.509549*wavelet-HHH_GLCM_Correlation+0.041439*wavelet-HHH_GLCM_InverseVariance+0.174459*original_firstorder_Minimum+0.599189*original_GLCM_Idn-0.309753*original_GLSZM_LargeAreaLowGrayLevelEmphasis-0.26451*wavelet-LLH_NGTDM_Strength+0.38903*wavelet-LHL_firstorder_Median-0.007962*wavelet-LHL_GLRLM_RunLengthNonUniformityNormalized+0.821409*wavelet-LHH_firstorder_Median-0.599362*wavelet-HLL_GLCM_MaximumProbability-0.543287*wavelet-HLL_GLSZM_LargeAreaLowGrayLevelEmphasis-0.906597*wavelet-HLH_firstorder_Mean+0.941644*wavelet-HHL_GLSZM_SizeZoneNonUniformityNormalized	the first 5 derived from CT, and the last 11 are PET features
Lesion_total4	1.030444-0.622254*log-sigma-3-0-mm-3D_firstorder_10Percentile+0.278994*log-sigma-3-0-mm-3D_GLCM_Idmn+0.720014*log-sigma-3-0-mm-3D_GLRLM_LowGrayLevelRunEmphasis-0.289411*wavelet-HLL_GLCM_MCC-0.567775*wavelet-HHH_firstorder_Mean-0.422396*wavelet-HHH_GLCM_Correlation-0.121343*wavelet-HHH_GLCM_InverseVariance+0.547775*original_firstorder_Minimum+0.064356*log-sigma-2-0-mm-3D_GLSZM_GrayLevelNonUniformityNormalized-0.214059*wavelet-LLH_GLCM_InverseVariance-1.044522*wavelet-HLL_GLSZM_LargeAreaLowGrayLevelEmphasis+0.507635*wavelet-HHH_GLCM_JointAverage-0.243426*wavelet-HHH_GLRLM_LowGrayLevelRunEmphasis-0.643355*wavelet-LLL_GLSZM_ LargeAreaLowGrayLevelEmphasis	the first 7 derived from CT, and the last 7 are PET features
Lesion_total5	1.096846-0.264878*log-sigma-3-0-mm-3D_firstorder_10Percentile+0.514756*log-sigma-3-0-mm-3D_GLRLM_LowGrayLevelRunEmphasis+0.297782*wavelet-HHH_GLCM_InverseVariance-0.282332*wavelet-LLL_GLSZM_LargeAreaLowGrayLevelEmphasis+0.672862*original_firstorder_Minimum-0.196907*log-sigma-2-0-mm-3D_GLSZM_SmallAreaLowGrayLevelEmphasis-0.322765*wavelet-LLH_GLCM_InverseVariance-0.276005*wavelet-LHL_GLSZM_LowGrayLevelZoneEmphasis-0.446075*wavelet-HLL_GLCM_MaximumProbability-0.580696*wavelet-HLL_GLSZM_LargeAreaLowGrayLevelEmphasis-0.43979*wavelet-HLH_GLCM_Imc2+0.653972*wavelet-HHH_firstorder_Median-0.574906*wavelet-LLL_GLSZM_SmallArea LowGrayLevel Emphasis	the first 4 derived from CT, and the last 9 are PET features
